# Nutrient supply systems and their effect on the performance of the Nile Tilapia (*Oreochromis niloticus*) and Lettuce (*Lactuca sativa*) plant integration system

**DOI:** 10.1038/s41598-024-54656-y

**Published:** 2024-02-20

**Authors:** El-Sayed Khater, Adel Bahnasawy, Heba Mosa, Wael Abbas, Osama Morsy

**Affiliations:** 1 Agricultural and Biosystems Engineering Department, Faculty of Agriculture, Benha University, P.O. Box 13736, Moshtohor, Toukh, Kalubia Egypt; 2 Basic and Applied Science Department, College of Engineering and Technology, Arab Academy for Science and Technology and Maritime Transport (AASTMT), P.O. Box 2033, Cairo, Egypt

**Keywords:** Aquaponic, Aquaculture, Hydroponic, Fish, Lettuce plant, Nutrients, Shoot, Root, Specific growth rate, Ecology, Environmental sciences, Engineering

## Abstract

The main aim of this work is to study the effect of different nutrient supply systems and their effect on the performance of the Nile Tilapia (*Oreochromis niloticus*) and Lettuce (*Lactuca sativa var. crispa*) plant integration system. To achieve that, five treatments having different culture systems (T1: Aquaculture (control), T2: Hydroponics (standard requirement: N = 210, P = 31, K = 234, Mg = 48, Ca = 200, S = 64, Fe = 14, Mn = 0.5, Zn = 0.05, B = 0.5, Cu = 0.02 and Mo = 0.01 ppm), T3: Aquaponics without nutrients addition, T4: Aquaponics with supplementary nutrients (KNO_3_, 101 g L^−1^, KH_2_PO_4_, 136 g L^−1^, Ca(NO_3_)_2_, 236 g L^−1^, MgSO_4_, 246 g L^−1^, K_2_SO_4_, 115 g L^−1^ and chelates for trace elements) in water (EC is 800 ppm) and T5: Aquaponics with supplementary nutrients spray on plants) were carried out. The previous systems were operated at three flow rates, namely, 1.0, 1.5 and 2.0 L h^−1^ plant^−1^. The various water quality parameters, plant growth and fish growth were studied. The result indicated that the highest values of N, P, k, Ca and Mg consumption rate were found with T2 and 1.5 L h^−1^ plant^−1^ of flow rate. The root length, fresh and dry of shoot and root for lettuce plants grown in T2 system was better than those grown in different culture system (T3, T4 and T5). Different culture systems showed significant effect on fish growth in terms of weight gain, specific growth rate and feed efficiency ratio. Higher growth rate was observed in treatment T3 as compared to other treatments. The production costs ranged from 2820.5 to 4885.4 LE ($ = 30.92 LE) for all culture systems.

## Introduction

Aquaculture is very important food resources and in the whole world. The world is about 63.6 million tons in the year^1^. Currently, many countries suffer a great deal from water scarcity. All aquaculture operation depends on the existence of water. Water losses during the aquaculture production stages cause environmental pollution due to it have several contaminants and nutrients^2^. Wastewater is used in aquaponics system mostly and the integration between fish and hydroponic is very useful. Hydroponic system is very important because it saves water and nutrients while plant uses the dissolved compounds in the wastewater^3,4^.

Hydroponics is the way of growing plants without soil, which use the nutrients and mineral solutions in a water solvent^5,6^. Aquaponics, is the combination between aquaculture and hydroponics, where, aquatic species grow together with the plant in soilless culture^7,8^.

Aquaponics has many advantages where compared with aquaculture and hydroponic, because it decreases the use of fertilizes and reduce the water treatments^8,9^. In this system, nutrients come out from fish and breakdown to be used by plants^10^. In the aquaponic system, nutrients are produced from fish outputs and are breakdown by the plants. Also, it does not use biofelter because the plants act as bio biofilter because the plants act as biofilters^11^. For these reasons, aquaponic system is considered economical, where; plant and fish integrate which benefit each other and the producer get two remarkable products. Aquaponic could be a strategy to solve the water consumption^12^ and prolong the life of water by decreasing the turnover rates and the environmental pollution, which in turn improve the return of production. Hydroponics produces a marketable plants and commercial aquatic species by using nutrient supply^2^.

Vegetables are the proper kind of plants that grow fast in hydroponic system, because of the waste water is rich with nutrients. These vegetables include lettuce, tomatoes and basil, these growing well on aquaculture wastewater^8,13^. In addition to herbal plants such as culinary, parsley, criterion and mint which grow fast in hydroponic and ensure high return compared to other crops such as cucumber, eggplants and okra^8,14^.

Nutrients concentrations depending on the ratio between the plant to fish, meanwhile, the nutritional values in normal growing plants system is very different compared to the soluble solution available to plant in hydroponic by fish^8^. The reduction rates of different nutrients differ very rapidly in the suboptimal concentrations and ratios of nutrients, which result in reduction in using solution for plants^7,15^.

In order to mitigate the global increasing of the utilization of chemical nutrients in plant production, using the nutrients of effluent water from fish farms is considered good unusual solution for this problem. This work aims to investigate the possibility of using different nutrient supply systems and their effect on the performance of the Nile Tilapia (*Oreochromis niloticus*) and Lettuce (*Lactuca sativa var. crispa*) plant integration system.

## Materials and methods

The experiment was conducted in the Fish Farms and Protected Houses Center, Faculty of Agriculture, Moshtohor, Benha University, Egypt (latitude 30° 21′ N and 31° 13′ E). During the period of November and December, 2022 season under the Regulations of Benha University which are consistent with the National and International guidelines and Legislation.

### Materials

#### The aquaponic system

**Figure 1:**
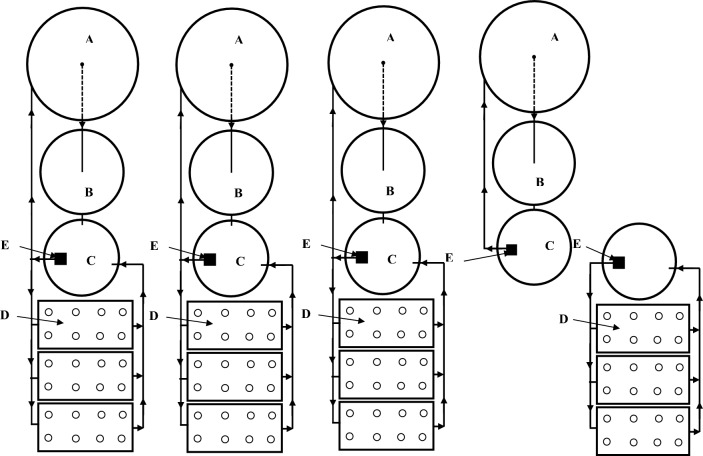
The experimental setup. (**A**) Fish tanks, (**B**) bio-sump tank, (**C**) water holding tank, (**D**) hydroponic units and (**E**) pump.

The system is a recirculate aquaponic system consists of fiberglass fish tanks, bio-sump tank, hydroponic units, pumps, air blower, water holding tank and reservoir, pipelines made of polyvinyl chloride (PE) were installed to connect the components of system (Fig. 1).

The fish tanks are cylindrical in shape and made of fiberglass with dimension of each tank are 1.0 m diameter and 1.0 m height. The capacity of each tank is 0.60 m^3^. The bio-sump tanks are made of fiberglass with dimension of 0.75 m diameter and 0.70 m height with a capacity of 0.25 m^3^, which are used in solid removal. These tanks have polyethylene sheets as a media to hold the bacteria (*Nitrosomonas* and *Nitrobacter*) from water.

Hydroponic unit include 12 tanks that made of polyethylene and rectangular in shape (dimensions of 80 × 40 × 30 cm for long, wide and high, respectively). The tanks were fixed on 1 m high from the ground with 2% slope and covered with foam board to fix the seedlings in it. Supplying irrigation water and solution to plants from water tank with the proper nutrient solution was carried out by 0.5 hp pump (Model First QB60—Head 25 m—Flow rate 30 L min^−1^—Power 0.5 hp, China).

The system has five circular polyethylene tanks with a 500 L capacity that used for collecting the drained solution by gravity from the ends of the systems. Air blower (Model C.C.P. Parma—Head 2.7 bar—Flow rate 10 m^3^ h^−1^—Power 1.0 kW, Italy) was used to supply the air to fish and hydroponic units, under various pressures through air stones.

The nutrient solutions that used in the experiment include: KNO_3_, 101 g L^−1^, KH_2_PO_4_, 136 g L^−1^, Ca(NO_3_)_2_, 236 g L^−1^, MgSO_4_, 246 g L^−1^, K_2_SO_4_, 115 g L^−1^ and chelates for trace elements into pre-acidified groundwater (from the following ppm concentration are achieved in this formulation: N = 210, P = 31, K = 234, Mg = 48, Ca = 200, S = 64, Fe = 14, Mn = 0.5, Zn = 0.05, B = 0.5, Cu = 0.02 and Mo = 0.01). EC and pH were further adjusted to 800–840 ppm and 6.0–6.5, respectively, after salt addition.

#### Fish and plant species

- Nile Tilapia fish (*Oreochromis niloticus*)
  Tilapia nilotica (*Oreochromis niloticus*) fish has an individual weight of 60 g. Each tank got 70 fish at the beginning of work. Weight of fish was taken each 10 days. The flow rate was adjusted according to the growth rate based on the calculation of flow rate required equal fish weight divided carrying capacity (The fish carrying capacity of a water body is the maximum fish yield that can be carried by the natural bait organisms in the water body under the ideal natural conditions without feeding and fertilization). Carrying capacity is determined according to Khater^16^. Flow rate for fish ranged from 205 to 420 L min^−1^. The daily feed rates at different fish sizes were carried out according to Rakocy^17^ with feed pellet diameter according to Jauncey and Ross^18^.
- Lettuce
  Lettuce seedlings were gown in the plastic cups (7 cm diameter and 7 cm height) filled with peat moss. The cups were irrigated daily using water with nutrient solution. Two weeks old lettuce seedlings were planted at 25.0 plant m^−2^ in the experimental tanks^19^. These seedlings were brought from the farm of Agriculture Faculty, Benha University.

### Methods

#### Treatments

In this study, five treatments include (T1: Aquaculture (control), T2: Hydroponics (standard requirement), T3: Aquaponics without nutrients addition, T4: Aquaponics with supplementary nutrients in water (EC is 800 ppm) and T5: Aquaponics with supplementary nutrients spray on plants) and three flow rate for hydroponic units were 1.0, 1.5 and 2.0 L h^−1^ plant^−1^. The experimental design was a split plot with four replicates.

#### Measurements

##### Water parameters

Water samples were taken at the influent and effluent of the system to measure pH, EC, temperature every day. Also, Total nitrogen (NH_3_, NO_2_ and NO_3_), Potassium, Phosphorus, Magnesium and Calcium were measured every 10 days at 10 am. EC Meter (Model ORION 230A—Range 0.0 to 19.99 ± 0.05, USA) was used to measured EC and temperature. pH Meter (Model ORION 105—Range 0.0 to 9999.9 ppm ± 0.5 ppm, USA) was used to measured pH. DO Meter (Model HANNA HI5421; Range: 0 to 90 mg L^−1^ ± 1.5%, Italy) was used to recorded dissolved oxygen. Spekol 11 (Model SPEKOL 11—Range 0. 1–1000 concentration ± 1 nm λ, UK) was used to measure ammonia, Nitrite and Phosphorus. Nitrate content was measured by using salicylic acid as described by Chapman^20^. Flame photometer (Model Jenway PFP7—Range 0. 1–999.9 ppm ± 0.2 ppm, USA) was used to measured Potassium, Calcium and Magnesium.

The nutrient consumption rate was determined using Eq. (1) according to ASAE^21^:

1 $$ NCR_{Nc} = \frac{{NCR_{in} - NCR_{out} }}{{\text{n}}} \times Q \, \times {24} $$

where NCR_Nc_ is the nutrients consumption rate, mg day^−1^ plant^−1^, NCR_in_ is the nutrients at inlet of the hydroponic unit, mg L^−1^, NCR_out_ is the nutrients at outlet of the hydroponic unit, mg L^−1^, Q is the discharge, L h^−1^, n is the number of plants

##### Plant parameters

Root length was measured every 10 days. The fresh and dry weight of shoot and root were measured at the end of the experiment. Dry weight the plants were measured by using oven dryer at 65 °C until constant weight was reached according to^22^.

##### Biological parameters of fish

Fish samples were taken every 10 days to determine the biological parameters which include: weight gain, specific growth rate, average daily gain, feed conversion ratio and feed efficiency ratio using the following equations:

2 $$ WG = W_{f} - W_{i} $$

3 $$ ADG = \frac{WG}{t} $$

4 $$ SGR = \frac{{\ln \,W_{f} - \ln \,W_{i} }}{t} \times 100 $$

5 $$ FCR = \frac{FI}{{WG \cdot n_{f} }} $$

6 $$ FER = \frac{{WG \cdot n_{f} }}{FI} $$

where WG is the weight gained, g; W_f_ is the mean final fish weight, g; W_i_ is the mean initial fish mass, g; ADG is the average daily gain, g day^−1^; SGR is the specific growth rate, % day^−1^ or g day^−1^; t is the time, day, FCR is the feed conversion ratio, g feed g^−1^ fish; FER is the feed Efficiency ratio, g fish g^−1^ feed; FI is the feed intake, g; n_f_ is the final number of fish in the tank.

### Cost calculation for the different systems

The costs were calculated according to Khater^23^. Table 1 shows the input parameters of calculate total production costs of tilapia fish and lettuce plant for different culture systems.

**Table 1:** The input parameters of calculate total production costs of tilapia fish and lettuce plant for different culture systems.

Cost item	Units	Culture system				
		T1	T2	T3	T4	T5
Fixed cost (LE)						
Culture units	LE	2000	940	2540	2540	2540
Pumps and fittings	LE	1550	1650	1800	1800	1800
Total fixed cost	LE	3550	2590	4340	4340	4340
Variable cost (LE)						
Fish fingerlings	LE	168	0	168	168	168
Lettuce seedlings	LE	0	18	18	18	18
Peat moss	LE	0	2.5	2.5	2.5	2.5
Labor	LE	50	50	50	50	50
Energy	LE	146.3	146.3	146.3	146.3	146.3
Fertilizers	LE	0	13.7	0	7.9	8.9
Feed	LE	151.7	0	151.7	151.7	151.7
Total variable cost	LE	516	230.5	536.5	544.4	545.4

Experiments and protocols were approved by the Research Committee in the Benha University. This study was carried out in compliance with the ARRIVE guidelines. This work is approved by the ethic committee at Benha University. All animal methods were carried out in accordance with relevant guidelines and regulations of Benha University. All the plant experiments and protocols were performed with relevant institutional, national, and international guidelines and legislation.

### Statistical analysis

The data were subjected to analysis using statistical package SPSS version 21 in which two ways ANOVA and Duncan Multiple Range Test were conducted at 5% significant levels and 95% confidence limit to know the significant differences between the means of different parameters.

## Results and discussion

### Water quality parameters

The water quality parameters for different culture systems (T1: Aquaculture (control), T2: Hydroponics (standard requirement), T3: Aquaponics without nutrients addition, T4: Aquaponics with supplementary nutrients in water (EC is 800 ppm) and T5: Aquaponics with supplementary nutrients spray on plants) during the growth period as shown in Table 2. Water temperatures are in the range of 25.37 ± 0.52 to 27.10 ± 0.36 °C for all culture systems under investigation. These systems have very close values of temperatures. Water temperature was within the range of 25.37 ± 0.52 to 27.10 ± 0.36 °C which is suitable for tilapia fish culture. Regarding the water pH, it varied within a range of 6.30 ± 0.23 to 6.91 ± 1.55, with no noticeable variations among the treatments. Different culture systems did not show any significant different on water pH which was found in desirable limits for fish as well as plant growth. Dissolved oxygen (DO) in water during the growth period varied within a range of 6.05 ± 0.89 to 6.51 ± 1.07 mg L^−1^ without significant variations during the growth period for the values of culture systems. The DO values fluctuated and varied nevertheless there were within narrow range and they were found within optimum limits for the fish as well as plant culture. Electrical conductivity (EC) values of water in Treatment T2 and T4 were significantly higher than that of T1, T3 and T5. EC values ranged from 671.44 ± 13.30 to 825.83 ± 19.05 mg L^−1^ for all systems under study.

**Table 2:** Water quality parameters.

Parameter	Treatments				
	T1	T2	T3	T4	T5
Temperature, °C	27.10 ± 0.36^a^	25.37 ± 0.52^a^	25.82 ± 0.41^a^	26.01 ± 0.29^a^	25.95 ± 0.34^a^
pH	6.91 ± 1.55^a^	6.30 ± 0.26^a^	6.74 ± 0.40^a^	6.66 ± 0.72^a^	6.61 ± 0.67^a^
EC, mg L^−1^	681.17 ± 20.56^a^	825.83 ± 19.05^b^	665.75 ± 15.99^a^	793.39 ± 11.07^b^	671.44 ± 13.30^a^
DO, mg L^−1^	6.51 ± 1.07^a^	6.05 ± 0.89^a^	6.57 ± 1.13^a^	6.30 ± 0.65^a^	6.62 ± 0.82^a^
NH_3_, mg L^−1^	0.025 ± 0.001^a^	–	0.024 ± 0.001^a^	0.023 ± 0.001^a^	0.021 ± 0.001^a^
Nitrite, mg L^−1^	0.39 ± 0.05^d^	–	0.16 ± 0.03^a^	0.29 ± 0.05^c^	0.25 ± 0.04^b^
Nitrate, mg L^−1^	14.85 ± 1.02^a^	163.55 ± 4.91^c^	9.44 ± 1.21^a^	146.96 ± 5.32^b^	12.50 ± 1.19^a^
Phosphorus, mg L^−1^	10.64 ± 2.06^b^	27.33 ± 2.38^d^	6.17 ± 1.55^a^	25.79 ± 2.50^c^	7.39 ± 1.39^a^
Potassium, mg L^−1^	43.80 ± 2.99^b^	201.73 ± 5.02^c^	36.24 ± 3.05^a^	195.34 ± 4.61^b^	37.05 ± 2.72^a^
Calcium, mg L^−1^	62.95 ± 2.65^c^	189.94 ± 5.33^f^	42.53 ± 2.87^a^	166.77 ± 5.83^d^	53.77 ± 3.01^b^
Magnesium, mg L^−1^	29.91 ± 2.11^a^	45.28 ± 3.17^b^	23.78 ± 2.90^a^	39.05 ± 2.66^b^	25.99 ± 1.88^a^

Means on the same row with different superscripts are significantly different (*p* < 0.05).

Regarding ammonia-N generation rate, the results reveal that ammonia values were higher in T1 and T3 system than other systems. Ammonia-N generation rate depends on the feeding rate in the system which was adjusted after each sampling as per the fish body weight. The lowest Nitrite-N concentration was observed in T3 (0.16 ± 0.03 mg L^−1^) as compared to T1, T4 and T5. Nitrate–N concentration (NO_3_–N) in T2 and T4 which were significantly higher than that of T1, T3 and T5 systems. Nitrate–N is relatively less toxic to fish and is not a health hazard except at exceedingly high levels above 300 mg L^−1^^24^. The phosphorus concentration values were 10.64 ± 2.06, 27.33 ± 2.38, 6.17 ± 1.55, 25.79 ± 2.50 and 7.39 ± 1.39 mg L^−1^ for T1, T2, T3, T4 and T5 culture systems, respectively. Phosphorus concentration was found significantly higher in the T2 and T4 system as compared to other systems. Potassium, calcium and magnesium concentrations also showed similar patterns.

### Nutrients consumption

Table 3 shows the nitrogen (N), phosphorus (P), potassium (K), calcium (Ca) and magnesium (Mg) consumption rate by lettuce plants during the growth period in different culture systems (T2: Hydroponics (standard requirement), T3: Aquaponics without nutrients addition, T4: Aquaponics with supplementary nutrients in water (EC is 800 ppm) and T5: Aquaponics with supplementary nutrients spray on plants) and different flow rates (1.0, 1.5 and 2.0 L h^−1^ plant^−1^). At 1.0 L h^−1^ flow rate, the nitrogen (N) consumption rates by lettuce plants values were 844.72 ± 21.35, 768.87 ± 19.88, 796.63 ± 19.23 and 808.84 ± 27.06 mg plant^−1^ for T2, T3, T4 and T5 systems during growth period, respectively. At 1.5 L h^−1^ flow rate, the nitrogen (N) consumption rates by lettuce plants values were 917.95 ± 29.08, 820.54 ± 26.01, 864.11 ± 25.54 and 880.39 ± 30.10 mg plant^−1^ for the previous systems, respectively. At 2.0 L h^−1^ flow rate, the nitrogen (N) consumption rates by lettuce plants values were 798.00 ± 19.16, 737.91 ± 17.77, 773.36 ± 18.69 and 781.95 ± 20.31 mg plant^−1^ for the previous systems, respectively.

**Table 3:** The nutrients consumption rate of lettuce plants grown in different culture systems.

Culture system	Flow rate, L h^−1^	Nutrients consumption rate, mg plant^−1^				
		N	P	K	Ca	Mg
T2	1.0	844.72 ± 21.35^ g^	779.56 ± 20.16^e^	2714.83 ± 56.08^d,e^	573.54 ± 20.61^ f,g^	459.11 ± 11.13^c^
	1.5	917.95 ± 29.08^j^	825.98 ± 25.32^ g^	2848.92 ± 67.76^f^	613.80 ± 17.01^ h^	482.54 ± 14.49^d,e^
	2.0	798.00 ± 19.16^d^	747.33 ± 15.49^c^	2580.81 ± 51.33^c^	540.61 ± 13.85^ c,d^	455.26 ± 10.37^b,c^
T3	1.0	768.87 ± 19.88^b^	726.81 ± 14.01^b^	2486.55 ± 52.55^b^	523.52 ± 15.39^b^	404.59 ± 9.97^a^
	1.5	820.54 ± 26.01^f^	739.09 ± 15.22^b,c^	2507.30 ± 49.80^b^	552.77 ± 16.66^e^	445.71 ± 12.22^b^
	2.0	737.91 ± 17.77^a^	697.74 ± 11.94^a^	2306.21 ± 47.73^a^	474.49 ± 15.08^a^	402.83 ± 13.00^a^
T4	1.0	796.63 ± 19.23^d^	754.55 ± 15.31^d^	2653.65 ± 51.63^d^	564.22 ± 17.23^f^	449.76 ± 13.97^b^
	1.5	864.11 ± 25.54^ h^	788.42 ± 17.79^f^	2676.12 ± 55.59^d^	601.68 ± 16.54^ h^	472.03 ± 12.03^d^
	2.0	773.36 ± 18.69^b^	726.29 ± 15.67^b^	2506.33 ± 46.67^b^	531.91 ± 13.31^c^	447.12 ± 10.23^b^
T5	1.0	808.84 ± 27.06^e^	761.07 ± 16.64^d^	2685.30 ± 57.30^d^	567.84 ± 14.43f.	452.69 ± 11.16^b^
	1.5	880.39 ± 30.10^i^	795.55 ± 19.27^f^	2689.87 ± 51.99^d^	606.81 ± 18.91^ h^	476.14 ± 12.63^d^
	2.0	781.95 ± 20.31^c^	732.26 ± 15.51^b^	2514.11 ± 49.07^b^	535.70 ± 15.08^c^	451.88 ± 11.85^b^

Means on the same column with different superscripts are significantly different (*p* < 0.05).

At 1.0 L h^−1^ flow rate, the phosphorus (P) consumption rates by lettuce plants values were 779.56 ± 20.16, 726.81 ± 14.01, 754.55 ± 15.31 and 761.07 ± 16.64 mg plant^−1^ for T2, T3, T4 and T5 systems during growth period, respectively. At 1.5 L h^−1^ flow rate, the phosphorus (P) consumption rates by lettuce plants values were 825.98 ± 25.32, 739.09 ± 15.22, 788.42 ± 17.79 and 795.55 ± 19.27 mg plant^−1^ for the previous systems, respectively. At 2.0 L h^−1^ flow rate, the phosphorus (P) consumption rates by lettuce plants values were 747.33 ± 15.49, 697.74 ± 11.94, 726.29 ± 15.67 and 732.26 ± 15.51 mg plant^−1^ for the previous systems, respectively.

At 1.0 L h^−1^ flow rate, the potassium (K) consumption rates by lettuce plants values were 2714.83 ± 56.08, 2486.55 ± 52.55, 2653.65 ± 51.63 and 2685.30 ± 57.30 mg plant^−1^ for T2, T3, T4 and T5 systems during growth period, respectively. At 1.5 L h^−1^ flow rate, the potassium (K) consumption rates by lettuce plants values were 2848.92 ± 67.76, 2507.30 ± 49.80, 2676.12 ± 55.59 and 2689.87 ± 51.99 mg plant^−1^ for the previous systems, respectively. At 2.0 L h^−1^ flow rate, the potassium (K) consumption rates by lettuce plants values were 2580.81 ± 51.33, 2306.21 ± 47.73, 2506.33 ± 46.67 and 2514.11 ± 49.07 mg plant^−1^ for the previous systems, respectively.

The calcium (Ca) consumption rate by lettuce plants values were 573.54 ± 20.61, 613.80 ± 17.01 and 540.61 ± 13.85, 523.52 ± 15.39, 552.77 ± 16.66 and 474.49 ± 15.08, 564.22 ± 17.23, 601.68 ± 16.54 and 531.91 ± 13.31 and 567.84 ± 14.43, 606.81 ± 18.91 and 535.70 ± 15.08 mg plant^−1^ for 1.0, 1.5 and 2.0 L h^−1^ plant^−1^, respectively, for T2, T3, T4 and T5 systems during growth period. The magnesium (Mg) consumption rate by lettuce plants values were 459.11 ± 11.13, 482.54 ± 14.49 and 455.26 ± 10.37, 404.59 ± 9.97, 445.71 ± 12.22 and 402.83 ± 13.00, 449.76 ± 13.97, 472.03 ± 12.03 and 447.12 ± 10.23 and 452.69 ± 11.16, 476.14 ± 12.63 and 451.88 ± 11.85 mg plant^−1^ for 1.0, 1.5 and 2.0 L h^−1^ plant^−1^, respectively, for T2, T3, T4 and T5 systems during growth period.

The results indicate that the highest values of N, P, k, Ca and Mg consumption rate were 917.95 ± 29.08, 825.98 ± 25.32, 2848.92 ± 67.76, 613.80 ± 17.01 and 613.80 ± 17.01 mg plant^−1^ were found with T2 and 1.5 L h^−1^ plant^−1^ of flow rate, while, the lowest values of N, P, k, Ca and mg consumption rate were 737.91 ± 17.77, 697.74 ± 11.94, 2306.21 ± 47.73, 474.49 ± 15.08 and 402.83 ± 13.00 mg plant^−1^ were found with T3 and 2.0 L h^−1^ plant^−1^ of flow rate. These results agreed with those obtained by^25,26^ whose found that the highest values of nutrients consumption rate of plant were found with a flow rate of 1.5 L h^−1^ plant^−1^.

The statistical analysis showed that the differences between the obtained data of nutrients consumption rate of lettuce plants due to the effect of culture system (A) and flow rate (B) were significant. The analysis showed also that the interaction between both AB was significant.

### Plant growth parameters

#### Root length

**Figure 2:**
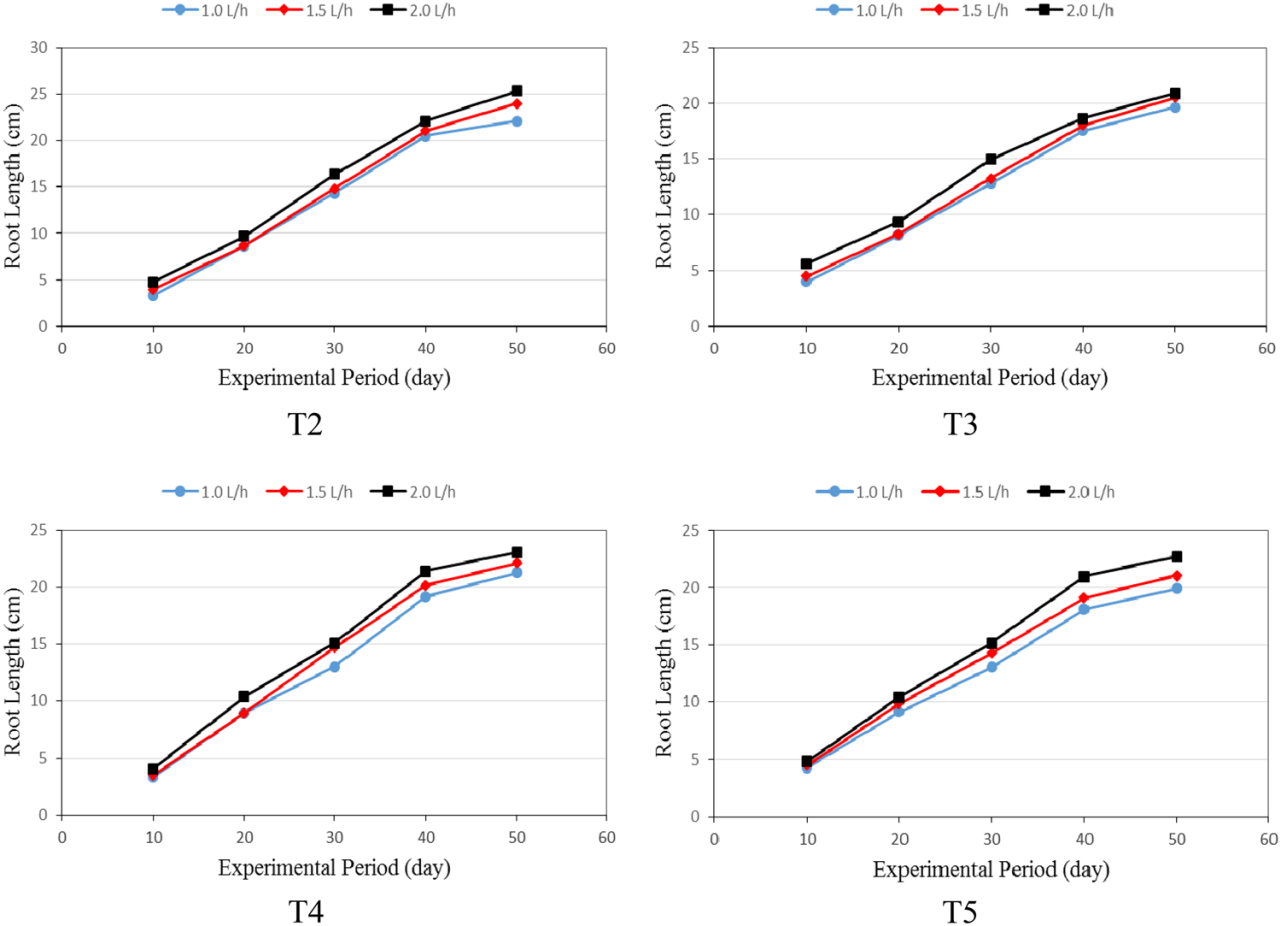
The root length of Lettuce plants grown in different culture systems.

The root length of lettuce plants grown in different culture systems (T2: Hydroponics (standard requirement), T3: Aquaponics without nutrients addition, T4: Aquaponics with supplementary nutrients in water (EC is 800 ppm) and T5: Aquaponics with supplementary nutrients spray on plants) and different flow rates for hydroponic units (1.0, 1.5 and 2.0 L h^−1^ plant^−1^) during the experimental period are shown in Fig. 2. It could be seen that the results indicate that the root of the lettuce plant grown in different culture system increases with increasing flow rate and plant age. It was noticed that there was not any overlapping (interference) between roots of the growing plants as a result of choosing a suitable distance (20 cm) apart between plants during different growth stages. If there is any overlapping existed it was very limited (not more than 5.0%). These results were in agreement with^16^ found that the plant spacing for lettuce was (20–25 cm).

The results also indicate that the root length for lettuce plants grown in T2 system was taller than those grown in different culture system (T3, T4 and T5). It could be seen that the highest value of root length was 25.32 ± 2.55 cm was found with T2 and 2.0 L h^−1^ plant^−1^ of flow rate, while, the lowest value of root length was 19.67 ± 1.86 cm was found with T3 and 1.0 L h^−1^ plant^−1^ of flow rate. Generally, the growth of plant in the hydroponic system depend on the optimum conditions and the amounts of nutrients available to root system and the oxygen available, which has relationship with the temperature and pressure. These results are in agreement with those obtained by^27^. Regarding the effect of flow rate, the results indicate that the root length increases with increasing flow rate. It which ranged from 3.28 ± 0.39 to 25.32 ± 2.55 cm depending on all treatments under study.

Multiple regression analysis was carried out to obtain a relationship between the root length of lettuce plants as dependent variable and different both of culture system, flow rate (1.0, 1.5 and 2.0 L h^−1^) and experimental period (1 to 50 day) as independent variables. The best fit for this relationship is presented in the following equation:

7 $$ For\quad T{2}\quad R{\text{L}}_{{2}} = - 3.74 + 0.52PA + 1.88Q \, \quad {\text{ R}}^{{2}} = 0.98 $$

8 $$ For\quad T{3}\quad R{\text{L}}_{{3}} = - 1.36 + 0.41PA + 1.46Q\quad {\text{R}}^{{2}} = 0.98 $$

9 $$ For\quad T{4}\quad R{\text{L}}_{{4}} = - 2.92 + 0.48PA + 1.65Q\quad {\text{R}}^{{2}} = 0.97 $$

10 $$ For\quad T{5}\quad R{\text{L}}_{{5}} = - 1.97 + 0.43PA + 1.93Q\quad {\text{R}}^{{2}} = 0.97 $$

where RL is the root length of lettuce plant, cm, PA is the lettuce plant age, day, Q is the flow rate, L h^−1^.

#### Fresh and dry weight of shoot

**Figure 3:**
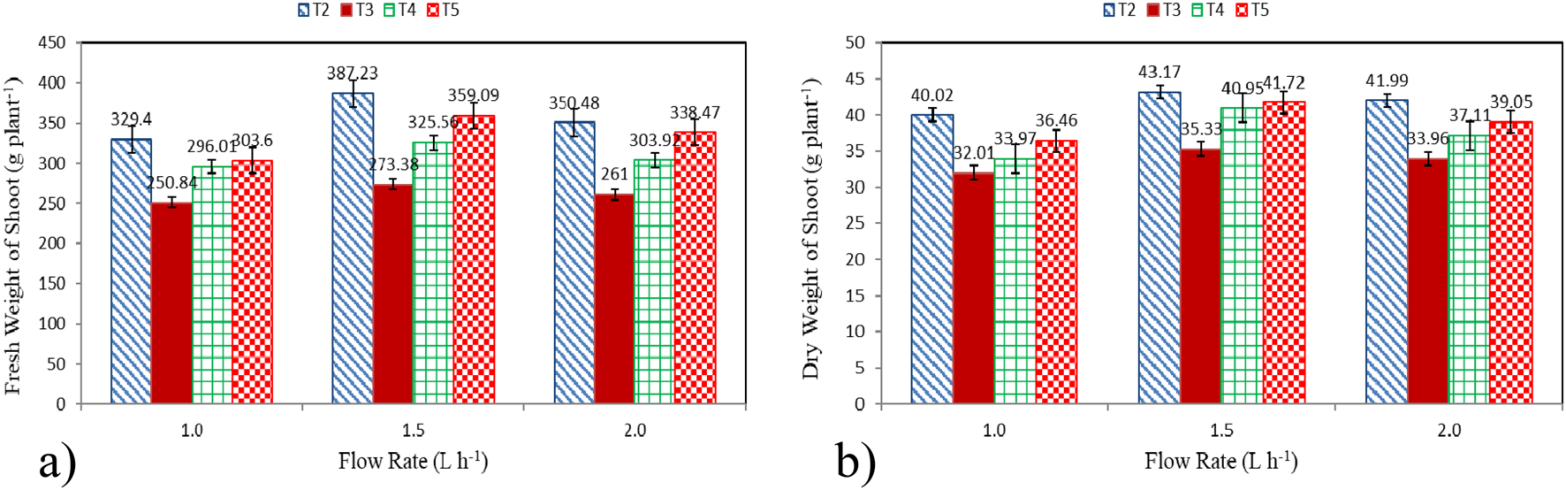
Fresh and dry weight of shoot of lettuce plants, (**a**) Fresh weight, and (**b**) Dry weight.

Fresh and dry weight of shoot of lettuce plants grown in different culture systems (T2: Hydroponics (standard requirement), T3: Aquaponics without nutrients addition, T4: Aquaponics with supplementary nutrients in water (EC is 800 ppm) and T5: Aquaponics with supplementary nutrients spray on plants) and different flow rates (1.0, 1.5 and 2.0 L h^−1^ plant^−1^) at the end of growth period (50 days) are shown in Fig. 3a,b. The results indicate that the fresh and dry weight of shoot of lettuce plants grown in T2 system were better than those of different culture system (T3, T4 and T5). It could be observed that the fresh weight of shoot of lettuce plants were 329.40 ± 12.91, 387.23 ± 13.08 and 350.48 ± 15.37, 250.84 ± 9.92, 273.38 ± 11.12 and 261.00 ± 9.86, 296.01 ± 15.54, 325.56 ± 10.01 and 303.92 ± 13.33 and 303.60 ± 8.95, 359.09 ± 10.13 and 338.47 ± 14.07 g plant^−1^ for 1.0, 1.5 and 2.0 L h^−1^ plant^−1^, respectively, for T2, T3, T4 and T5 systems at the end of growth period. While, the dry weight of shoot of lettuce plants were 40.02 ± 1.57, 43.17 ± 2.09 and 41.99 ± 2.11, 32.01 ± 0.99, 35.33 ± 1.44 and 33.96 ± 1.72, 33.97 ± 1.60, 40.95 ± 1.27 and 37.11 ± 3.15 and 36.46 ± 0.98, 41.72 ± 2.13 and 39.05 ± 3.00 g plant^−1^ for 1.0, 1.5 and 2.0 L h^−1^ plant^−1^, respectively, for T2, T3, T4 and T5 systems at the end of growth period. The variations in growth and yield of lettuce plants are explained by the variation of nutrients available and flow rates. Generally, the growth of lettuce is dependent on the nutrients available under proper conditions of temperature and pressure besides the balance of nutrients and oxygen supply. These results were in agreement with^28^.

The results indicate that the highest values of fresh and dry weight of shoot (387.23 ± 13.08 and 43.17 ± 2.09 g plant^−1^) were found with T2 and 1.5 L h^−1^ plant^−1^ of flow rate, while, the lowest values of fresh and dry weight of shoot (250.84 ± 9.92 and 32.01 ± 0.99 g plant^−1^) were found with T3 and 1.0 L h^−1^ plant^−1^ of flow rate. These results agreed with those obtained by^25,26,29^ whose found that the highest values of fresh and dry weight of plant were found with a flow rate of 1.5 L h^−1^ plant^−1^.

#### Fresh and dry weight of root

**Figure 4:**
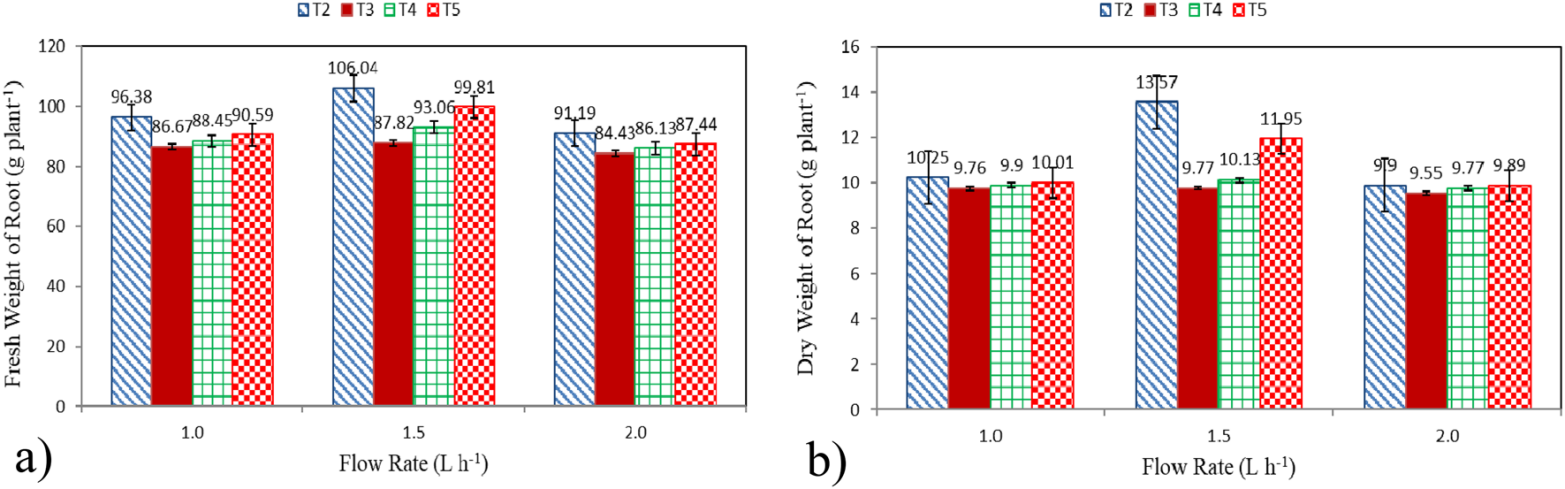
Fresh and dry weight of root of lettuce plants, (**a**) Fresh weight, and (**b**) Dry weight.

Fresh and dry weight of root of lettuce plants grown in different culture systems (T2: Hydroponics (standard requirement), T3: Aquaponics without nutrients addition, T4: Aquaponics with supplementary nutrients in water (EC is 800 ppm) and T5: Aquaponics with supplementary nutrients spray on plants) and different flow rates (1.0, 1.5 and 2.0 L h^−1^ plant^−1^) at the end of growth period (50 days) are shown in Fig. 4a,b. The results indicate that the fresh and dry weight of root of lettuce plants grown in T2 system were better than those of different culture system (T3, T4 and T5). It could be observed that the fresh weight of root of lettuce plants were 96.38 ± 4.81, 106.04 ± 5.24 and 91.19 ± 3.80, 86.67 ± 3.99, 87.82 ± 3.64 and 84.43 ± 4.05, 88.45 ± 4.82, 93.06 ± 3.77 and 86.13 ± 2.93 and 90.59 ± 4.76, 99.81 ± 4.01 and 87.44 ± 5.07 g plant^−1^ for 1.0, 1.5 and 2.0 L h^−1^ plant^−1^, respectively, for T2, T3, T4 and T5 systems at the end of growth period. While, the dry weight of root of lettuce plants were 10.25 ± 0.88, 13.57 ± 1.22 and 9.90 ± 0.69, 9.76 ± 0.83, 9.77 ± 1.33 and 9.55 ± 0.94, 9.90 ± 1.03, 10.13 ± 0.89 and 9.77 ± 1.00 and 10.01 ± 0.77, 11.95 ± 1.21 and 9.89 ± 0.64 g plant^−1^ for 1.0, 1.5 and 2.0 L h^−1^ plant^−1^, respectively, for T2, T3, T4 and T5 systems at the end of growth period. These findings may be referred to that the plants in hydroponic system (T2) are grown at precise control over the nutrient solution and ability of them to be in their most favorable growing condition. These results were in agreement with^30,31^.

### Fish growth parameters

#### Fish weight

**Figure 5:**
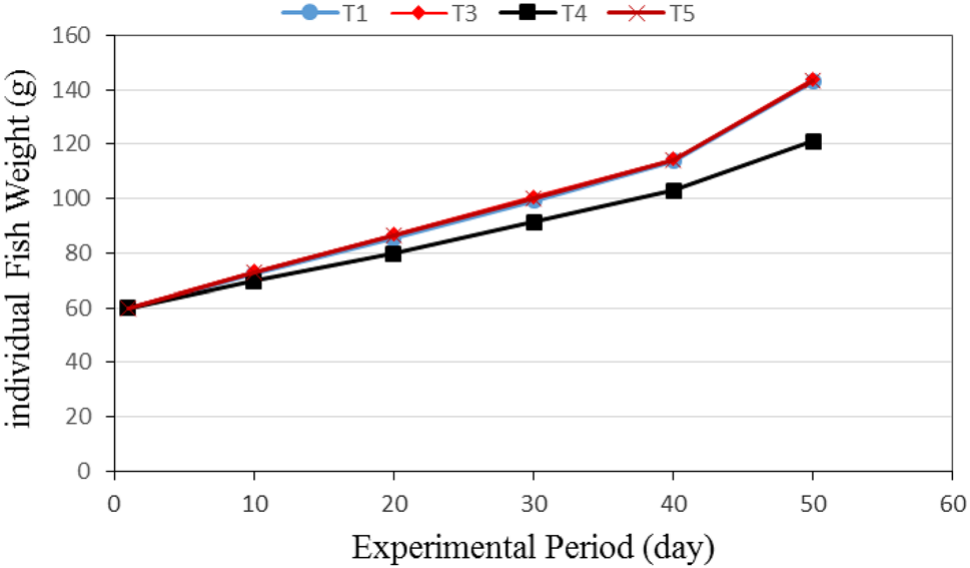
Individual fish weight for different culture systems.

Figure 5 shows the individual fish weight for different culture systems (T1: Aquaculture (control), T3: Aquaponics without nutrients addition, T4: Aquaponics with supplementary nutrients in water (EC is 800 ppm) and T5: Aquaponics with supplementary nutrients spray on plants) during the experimental period. The results indicate that the individual fish weight in different culture system increases with increasing experimental period. It could be seen that the individual fish weight significantly increased from 60.00 ± 1.28 to 143.06 ± 2.09, 60.00 ± 1.28 to 144.08 ± 2.44, 60.00 ± 1.28 to 121.01 ± 1.97 and 60.00 ± 1.28 to 143.49 ± 3.51 g, when the growth period increased from 1 to 50 days, respectively, for T1, T3, T4 and T5 systems. The results also indicate that the highest value of individual fish weight (144.08 ± 2.44 g) was found for T3 culture system, while, the lowest value of individual fish weight (121.01 ± 1.97 g) was found for T4 culture system.

#### Biological fish parameters

Table 4 shows the fish average daily growth, specific growth rate, feed conversion ratio and feed efficiency ratio for different culture systems (T1: Aquaculture (control), T3: Aquaponics without nutrients addition, T4: Aquaponics with supplementary nutrients in water (EC is 800 ppm) and T5: Aquaponics with supplementary nutrients spray on plants) at the end growing period. It could be seen that the results indicate that the average daily growth (ADG) were 1.66 ± 0.44, 1.68 ± 0.40, 1.22 ± 0.23 and 1.67 ± 0.40 g day^−1^ for T1, T3, T4 and T5 systems, respectively, at the end of growth period. The specific growth rate (SGR) was 1.74 ± 0.19, 1.75 ± 0.26, 1.40 ± 0.11 and 1.74 ± 0.25% day^−1^ for T1, T3, T4 and T5 systems, respectively, at the end of growth period. The feed conversion ratio (FCR) was 1.46 ± 0.16, 1.48 ± 0.17, 1.58 ± 0.18 and 1.49 ± 0.16 g feed g^−1^ fish for T1, T3, T4 and T5 systems, respectively, at the end of growth period. The feed efficiency ratio (FER) was 0.98 ± 0.07, 0.96 ± 0.05, 0.86 ± 0.04 and 0.95 ± 0.06 g fish g^−1^ feed for T1, T3, T4 and T5 systems, respectively, at the end of growth period.

**Table 4:** Fish performance and feed utilization parameters.

Parameter	Culture systems			
	T1	T3	T4	T5
ADG, g day^−1^	1.66 ± 0.44^b^	1.68 ± 0.40^b^	1.22 ± 0.23^a^	1.67 ± 0.40^b^
SGR, % day^−1^	1.74 ± 0.19^b^	1.75 ± 0.26^b^	1.40 ± 0.11^a^	1.74 ± 0.25^b^
FCR, g feed g^−1^ fish	1.46 ± 0.16^a^	1.48 ± 0.17^a^	1.58 ± 0.18^b^	1.49 ± 0.16^a^
FER, g fish g^−1^ feed	0.98 ± 0.07^b^	0.96 ± 0.05^b^	0.86 ± 0.04^a^	0.95 ± 0.06^b^

Means on the same row with different superscripts are significantly different (*p* < 0.05).

The results indicate that the highest values of the fish growth rate, specific growth rate and feed efficiency ratio were 1.68 ± 0.40 g day^−1^, 1.75 ± 0.26% day^−1^ and 0.96 ± 0.05 g fish g^−1^ feed, respectively, were found for T3 culture system, while, the highest value of the feed conversion ratio was 1.58 ± 0.18 g feed g^−1^ fish was found for T4 culture system. On the other hand, the lowest values of the fish growth rate, specific growth rate and feed efficiency ratio were 1.22 ± 0.23 g day^−1^, 1.40 ± 0.11% day^−1^ and 0.86 ± 0.04 g fish g^−1^ feed, respectively, were found for T4 culture system, while, the highest value of the feed conversion ratio was 1.46 ± 0.16 g feed g^−1^ fish was found for T1 culture system. Better fish growth performance in T3 may be as a result of better water quality parameters. These results were in agreement with^32,33^.

From statistical analysis of the experimental data, there were no significant different between T1, T3 and T5 systems on the fish growth rate, specific growth rate, feed conversion ratio and feed efficiency ratio, meanwhile, there were significant differences among T4 system and T1, T3 and T5 systems on the fish growth rate, specific growth rate, feed conversion ratio and feed efficiency ratio.

### Production costs

Table 5 shows the fixed, variable and total costs of tilapia fish and lettuce plant for different culture systems (T1: Aquaculture (control), T3: Aquaponics without nutrients addition, T4: Aquaponics with supplementary nutrients in water (EC is 800 ppm) and T5: Aquaponics with supplementary nutrients spray on plants) at the end growing period. It could be seen that the fixed costs were 3550, 2590, 4340, 4340 and 4340 LE for T1, T2, T3, T4 and T5 culture systems, respectively. The variable costs were 516, 230.5, 536.5, 544.4 and 545.4 LE for T1, T2, T3, T4 and T5 culture systems, respectively. The total production costs were 4066, 2820.5, 4876.5, 4884.4 and 4885.4 LE for T1, T2, T3, T4 and T5 culture systems, respectively. From statistical analysis of the experimental data, there were no significant different between T3, T4 and T5 systems on the total production costs.

**Table 5:** The total production costs of tilapia fish and lettuce plant for different culture systems.

Cost item	Culture system				
	T1	T2	T3	T4	T5
Fixed costs, L.E	3550^b^	2590^a^	4340^c^	4340^c^	4340^c^
Variable costs, L.E	516^b^	230.5^a^	536.5^c^	544.4^c^	545.4^c^
Total costs, L.E	4066^b^	2820.5^a^	4876.5^c^	4884.4^c^	4885.4^c^

Means on the same row with different superscripts are significantly different (*p* < 0.05).

## Conclusions

From this study, it is concluded that the highest values of N, P, k, Ca and Mg consumption rate were found with hydroponic standard requirement (T2) and 1.5 L h^−1^ plant^−1^ of flow rate, while, the lowest values of N, P, k, Ca and Mg consumption rate were found with aquaponics without nutrients additions (T3) and 2.0 L h^−1^ plant^−1^ of flow rate. The root length for lettuce plants grown in T2 system was taller than those grown in different culture system (T3, T4 and T5). The fresh and dry of shoot and root of lettuce plants grown in T2 system were better than those of different culture system (T3, T4 and T5). The highest value of individual fish weight was found for T3 culture system, while, the lowest value of individual fish weight was found for T4 culture system. The highest values of the fish growth rate, specific growth rate and feed efficiency ratio were found for T3 culture system, while, the highest value of the feed conversion ratio was found for T4 culture system. The production costs ranged from 2820.5 to 4885.4 LE ($ = 30.92 LE) for all culture systems. Finally, to integrate between fish and plant, you must adjust and monitor the nutritional elements requirements and the flow rate to give both fish and plants their optimum requirements to get higher yield of both of them. Further studies are recommended to adjust the environmental factors the proposed system.

## Data Availability

The datasets used and/or analyzed during the current study available from the corresponding author on reasonable request.
